# A survey across orbital lymphoma in Poland: Multicenter retrospective study of polish lymphoma research group (PLRG)

**DOI:** 10.1002/cam4.5223

**Published:** 2022-09-26

**Authors:** Elżbieta Kalicińska, Agnieszka Giza, Jan Maciej Zaucha, Sebastian Giebel, Dagmara Zimowska‐Curyło, Iga Andrasiak, Wojciech Spychałowicz, Jerzy Wojnar, Andrzej Balcerzak, Joanna Romejko‐Jarosińska, Ewa Paszkiewicz‐Kozik, Wanda Knopińska‐Posłuszny, Justyna Rybka, Paula Jabłonowska, Tomasz Wróbel

**Affiliations:** ^1^ Department of Hematology, Blood Neoplasms and Bone Marrow Transplantation Wrocław Medical University Wrocław Poland; ^2^ Department of Hematology Jagiellonian University Medical College Kraków Poland; ^3^ Department of Hematology and Transplantology Medical University of Gdańsk Gdańsk Poland; ^4^ Department of Bone Marrow Transplantation and Oncohematology Maria Sklodowska‐Curie National Research Institute of Oncology Gliwice Poland; ^5^ Independent Researcher Wrocław Poland; ^6^ Department of Internal Medicine and Oncology Silesian Medical University Katowice Poland; ^7^ Department of Hematology and Bone Marrow Transplantation Poznan University of Medical Science Poznań Poland; ^8^ Maria Sklodowska‐Curie National Research Institute of Oncology Warsaw Poland; ^9^ Department of Hematology and Bone Marrow Transplantation Maritime Hospital Gdynia Poland

**Keywords:** head and neck cancer, MALT, Ocular adnexal lymphoma, prognosis

## Abstract

**Objective:**

To investigate the prevalence of histopathological subtypes, the clinical stage at presentation and treatment modalities in Polish patients with orbital lymphoma (OL) and to determine prognostic outcomes.

**Methods:**

The retrospective study of 107 patients with OL treated in a 14‐year period in Polish hematological centers. The analysis included histopathological subtype, disease clinical advancement, treatment modalities, progression‐free survival (PFS), and overall survival (OS).

**Results:**

The median patient age was 60 years (range 51–71). Mucosa‐associated lymphoid tissue (MALT) lymphoma accounted for slightly more than half of all cases of orbital lymphoma (51%). The second most common subtype was diffuse large B‐cell lymphoma (DLBCL) (29%). Primary orbital lymphoma was diagnosed in 48% of all patients. According to the Ann Arbor, localized stage IE of orbital lymphoma was diagnosed only in 39% of all patients. Systemic involvement was observed in more than half of all patients (52%). The median follow‐up period was 30 months (range 0–160 months). Patients with non‐MALT lymphoma had a significantly inferior PFS compared to patients with MALT lymphoma, (*p* = 0.047). Patients with primary orbital lymphoma had a superior PFS compared to patients with secondary orbital lymphoma [median PFS 104.5 months vs. 33.4 months], (*p* = 0.069).

Younger patients with MALT lymphoma were characterized by superior PFS (median PFS not reached) compared to other studied subgroups of patients (older patients with MALT lymphoma, younger and older non‐MALT lymphoma patients) with a median PFS of 30.5, 32.2, 32.6 months respectively (*p* = 0.039). Patients treated with chemotherapy alone had inferior PFS compared to patients treated with combined therapies (*p* = 0.034). The median PFS across patients who received chemotherapy alone was 23.7 months, whereas across other patients was 73.9 months.

**Conclusions:**

Secondary lymphoma accounts for more than half of the orbital lymphoma in Polish population. The advanced clinical stage of the disease (non‐IE according to Ann Arbor) concerns two‐thirds of the overall population of patients with orbital lymphomas and one‐third of MALT lymphoma patients. The high incidence of advanced stages of orbital lymphoma may indicate the need for combined treatment. Combined orbital lymphoma treatment is associated with superior PFS compared to chemotherapy alone in overall population of patients with orbital lymphoma.

## Graphical abstract

The advanced clinical stage of the disease (non‐IE according to the Ann Arbor) concerns two thirds of the overall population of patients with ocular adnexal lymphoma and one third of MALT lymphoma patients. Due to the high prevalence of advanced stages of ocular adnexal lymphoma, treatment with combined therapies is warranted. Combined treatment tends to improve PFS compared to chemotherapy alone in the overall population of patients with ocular adnexal lymphoma.

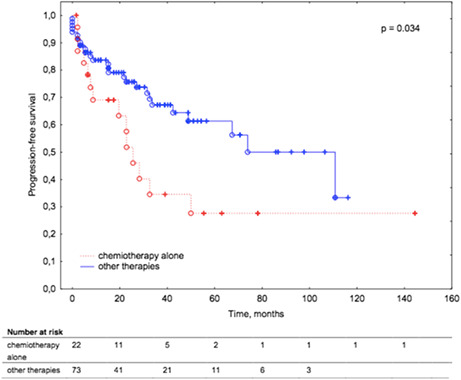

## 1 INTRODUCTION

Orbital lymphoma constitutes for approximately 1%–2% of all non‐Hodgkin lymphoma and comprises over the half of all orbital tumors.^1^, ^2^, ^3^ The majority of orbital lymphoma are B‐cell origin and the extranodal marginal zone B‐cell lymphoma is the most common subtype.^2^ The prognosis of orbital lymphoma depends on the histopathological subtype and clinical stage.

Previous studies have emphasized the importance of racial and geographical differences in the clinical symptoms and severity of orbital lymphoma,^1^ which may influence the treatment methods.

Although some reports have shown no difference in the survival in non‐gastric MALT lymphoma patients receiving different therapies (either as monotherapy or in combination),^5^, ^6^, ^7^ currently due to reports of a high incidence of systemic involvement in orbital lymphoma,^4^ the analysis of the impact of the initial treatment (combined therapy vs. monotherapy) on PFS and OS in patients with newly diagnosed orbital lymphoma seems to be important.

Some studies indicate that radiotherapy alone may be associated with a high risk of orbital lymphoma recurrence.^8^, ^9^ Additionally, a few authors have suggested that a combination treatment with chemotherapy is beneficial in orbital lymphoma.^10^

However, due to the belief that the majority of orbital lymphoma has a limited stage of advancement, the importance of combined treatment in these patients has not yet been investigated.

The aim of our study was to analyze the prevalence of histopathological subtypes of orbital lymphoma, the stage of clinical advancement at the time of initial diagnosis, treatment modalities, and their impact on the progression‐free survival and outcome in a cohort of Polish patients.

## 2 MATERIALS AND METHODS

### 2.1 Study population

We retrospectively analyzed 107 patients with orbital lymphoma, hospitalized in eight Polish medical centers between January 1998 and December 2012, including 55 patients with MALT orbital lymphoma. Data were collected from the local medical records.

Histopathological diagnosis was based on the REAL (Revised European‐American Lymphoma) classification for lymphoma diagnosed between 1998 and 2000. Lymphomas diagnosed between 2001 and 2007 were classified according to the WHO classification from 2001. All specimens obtained after 2008 were classified according to the 4th edition of the WHO classification from 2008.

The study was performed in accordance with the local ethics committee. The approval of local ethics committee was not obtained prior to initiating study because of its retrospective character. The collected data did not include any personally identifiable information therefore informed consent from the patients was not required.

### 2.2 Study design

Patients were divided into two groups: (1) with MALT orbital lymphoma, and (2) with non‐MALT orbital lymphoma. The non‐MALT lymphoma group included patients with other B‐lymphomas (DLBCL, FL, MCL) and patients with T/NK cell lymphomas. Patient's characteristics included: age, gender, symptoms, systemic involvement according to the Ann Arbor staging classification, type of treatment, response to applied treatment, progression‐free survival (PFS), and overall survival (OS). PFS was defined as the date of diagnosis to either the date of first relapse or progression after initial treatment, the date of death by any cause, or the date of last contact. OS was defined as the date of diagnosis to death by any cause or to the last follow‐up. The median follow‐up period was 30 months (range 0–160 months).

### 2.3 Statistical analyses

Statistical analyses were performed using R, version 4.0.5; the R Foundation for Statistical Computing, Vienna, Austria; and Statistica, version 13.1; TIBCO Software Inc., Palo Alto, California, USA. Endpoints were visualized by Kaplan–Meier plots and life tables. Categorical variables were presented as frequencies with percentages, whereas median and interquartile range (IQR) were used to describe continue variables. Groups were compared using log‐rank test for survival outcomes and *χ* ^2^ test for categorical variables. All statistical tests were two‐tailed with the significance level set at *p* = 0.05.

## 3 RESULTS

### 3.1 Clinical characteristics of patients with orbital lymphoma

The baseline clinical characteristics of 107 patients included in the analysis are given in Table 1.

**TABLE 1:** Baseline characteristics of 107 patients with orbital lymphoma

Variable	All patients, *n* (%)	MALT lymphoma	All non‐MALT lymphoma	Non‐MALT lymphoma histopathological subtypes				
				DLBCL	MCL	FL	Other B	T/NK‐cell lymphoma
No of patients, *n* (%)	107 (100)	55 (51)	52 (49)	31 (29)	8 (7)	4 (4)	3 (3)	6 (6)
Male, *n* (%)	39 (36)	18 (33)	21 (40)	14 (45)	2 (25)	0 (0)	0 (0)	5 (83)
Female, *n* (%)	68 (64)	37 (67)	31 (60)	17 (55)	6 (75)	4 (100)	3 (100)	1 (17)
Age median, years	60 (51–71)	62 (51–72)	60 (51–70)	64 (52–72)	56 (50–70)	54 (50–61)	71 (66–73)	47 (43–54)
Ann Arbor stage, *n* (%)								
IE	42 (39)	38 (69)	4 (8)	2 (6)	0 (0)	0 (0)	0 (0)	2 (33)
IIE	10 (9)	6 (11)	4 (8)	4 (13)	0 (0)	0 (0)	0 (0)	0 (0)
IIIE	7 (7)	2 (4)	5 (9)	1 (3)	1 (13)	1 (25)	1 (33)	1 (17)
IV	48 (45)	9 (16)	39 (75)	24 (77)	7 (88)	3 (75)	2 (67)	3 (50)
B symptoms, *n* (%)	27 (25)	3 (5)	24 (46)	13 (42)	6 (75)	1 (25)	2 (67)	2 (33)
Treatment								
Immunochemotherapy, *n* (%)	33 (31)	12 (22)	21 (40)	17 (55)	2 (25)	1 (25)	0 (0)	1 (17)
Chemotherapy alone, *n* (%)	25 (23)	14 (25)	11 (21)	2 (6)	4 (50)	2 (50)	1 (33)	2 (33)
Surgery alone, *n* (%)	12 (11)	12 (22)	0 (0)	0 (0)	0 (0)	0 (0)	0 (0)	0 (0)
Surgery combined with chemotherapy, *n* (%)	6 (6)	2 (4)	4 (8)	3 (10)	0 (0)	1 (25)	0 (0)	0 (0)
Radiotherapy alone, *n* (%)	6 (6)	5 (9)	3 (6)	1 (3)	0 (0)	0 (0)	0 (0)	0 (0)
Radiotherapy combined with chemotherapy, *n* (%)	18 (17)	5 (9)	11 (21)	7 (22)	2 (25)	0 (0)	1 (33)	3 (50)
Other (surgery with rituximab), *n* (%)	7 (7)	5 (9)	2 (4)	1 (3)	0 (0)	0 (0)	1 (33)	0 (0)

The majority of patients (64%) were females. Just over ahalf of patients (51%) had MALT lymphoma. Among remaining B type lymphoma, DLBCL was diagnosed in 29% of patients. Other B type lymphoma included: mantle cell lymphoma, follicular lymphoma, small lymphocytic lymphoma. Only 6% of patients had T‐cell/NK‐cell lymphoma. Primary lymphoma was identified in 48% of all patients. The majority of primary orbital lymphoma (stage IE or IIE) was diagnosed as MALT lymphoma (80%), whereas non‐MALT lymphoma was diagnosed mainly as secondary lymphoma (stage IIIE or IV) with *p* < 0.001.

B symptoms were more common in non‐MALT compared to MALT lymphoma (46% vs. 5%, *p* < 0.001). The most common symptoms among all patients were an objective mass (89%), proptosis (17%), limited motility (15%), and edema (14%). All symptoms are included in Table 2.

**TABLE 2:** Clinical manifestations of 107 patients with orbital lymphoma

Symptom	All patients, *n* (%)	MALT lymphoma	Non‐MALT lymphoma				
			DLBCL	MCL	FL	Other B	T/NK‐cell lymphoma
Proptosis	18 (17)	11 (20)	4 (13)	2 (25)	1 (25)	0 (0)	0 (0)
Mass	95 (89)	45 (82)	30 (97)	8 (100)	4 (100)	3 (100)	5 (83)
Edema	15 (14)	9 (16)	3 (10)	2 (25)	0 (0)	0 (0)	1 (17)
Photophobia	4 (4)	2 (4)	2 (6)	0 (0)	0 (0)	0 (0)	0 (0)
Limited motility	16 (15)	7 (13)	5 (16)	3 (38)	0 (0)	0 (0)	1 (17)
Blindness	5 (5)	1 (2)	4 (13)	0 (0)	0 (0)	0 (0)	0 (0)
Diplopia	9 (8)	6 (11)	2 (6)	0 (0)	0 (0)	1 (33)	0 (0)
Displacement	3 (3)	0 (0)	3 (10)	0 (0)	0 (0)	0 (0)	0 (0)
Dizziness	3 (3)	1 (2)	0 (0)	1 (13)	0 (0)	0 (0)	1 (17)

Among reported clinical signs, the presence of the objective mass was significantly more frequent in patients with non‐MALT compared to MALT lymphoma (96% vs 82%, *p* = 0.019). Other signs and symptoms such as proptosis, edema, displacement, photophobia, limited motility, diplopia, and dizziness occurred with similar frequency in both (MALT and non‐MALT lymphoma) studied groups of patients.

Sixty patients (56%) had unilateral disease and nine patients (8%) had bilateral disease. Data on laterality of the disease were missing in 38 patients (35%). Considering the anatomical location of the lymphoma, the most common location was the orbit in 40 patients (37%), followed by conjunctiva in 13 patients (12%) and eyelid in seven (7%) patients. Twelve patients in our cohort had involvement of more than one structure. Six (6%) had involvement of orbit and eyelid, four (4%) had involvement of the orbit and conjunctiva, and two (2%) had involvement of the eyelid and conjunctiva. No data on the anatomical localization of the lymphoma were available in 35 patients (33%).

The majority of patients (77%) were treated with chemotherapy (both alone and in combination with other therapies). Patients with non‐MALT lymphoma were significantly more often treated with immunochemotherapy compared to MALT lymphoma patients (40% vs. 22%, *p* = 0.038). Surgery alone was performed significantly more often in MALT lymphoma versus non‐MALT lymphoma patients (22% vs. 0%, *p* < 0.001). Radiotherapy combined with immunochemotherapy or chemotherapy was applied more often in non‐MALT lymphoma compared to MALT lymphoma (21% vs. 9%, *p* = 0.028). Complete response after first‐line treatment was achieved in 77% of all studied patients.

The median follow‐up was 30 months (range 0–160 months).

### 3.2 Treatment outcome and survival in patients with orbital lymphoma

**FIGURE 1:**
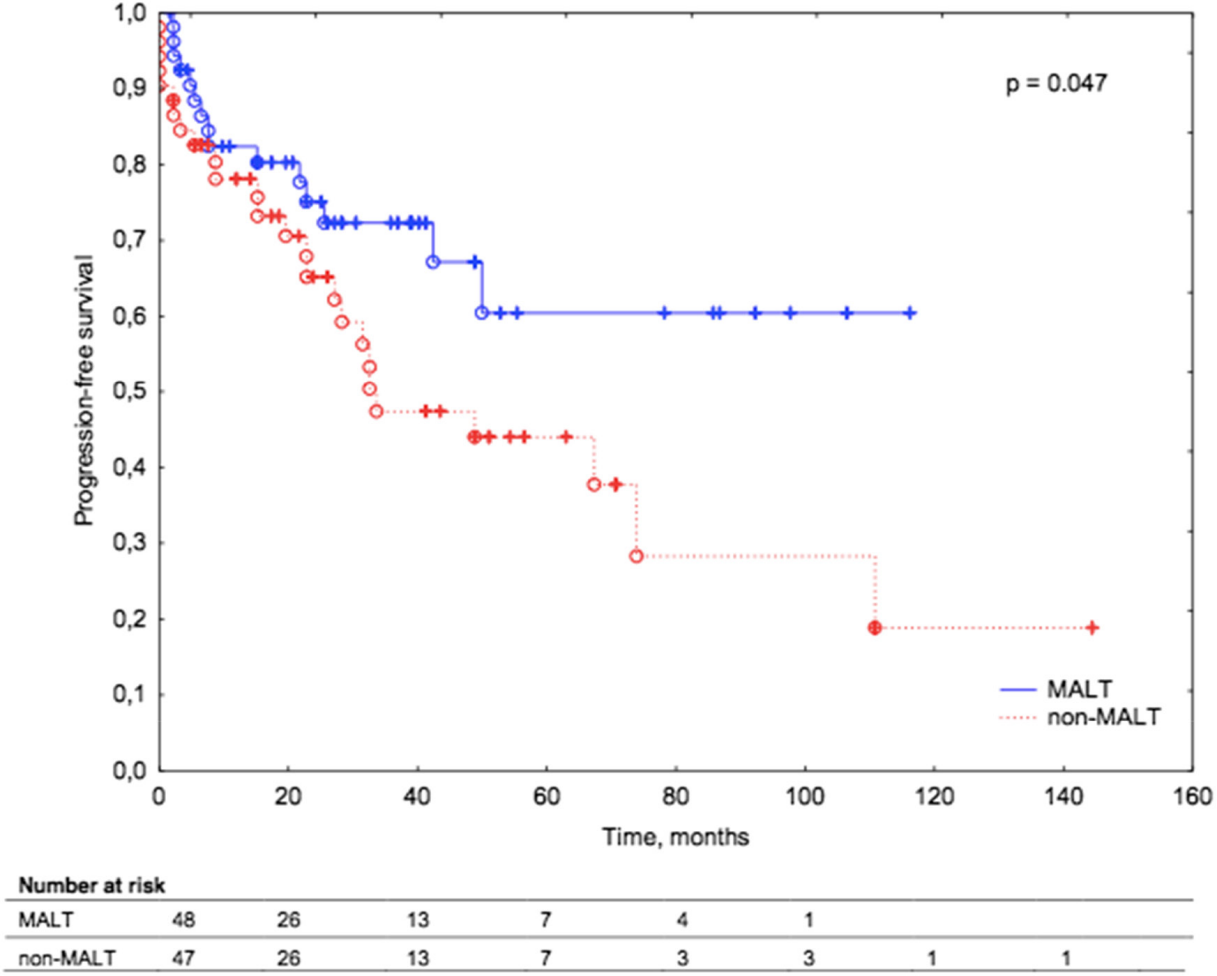
Kaplan–Meier survival curves for progression‐free survival in patients with ocular adnexal lymphoma according to lymphoma subtype (MALT versus non‐MALT).

**FIGURE 2:**
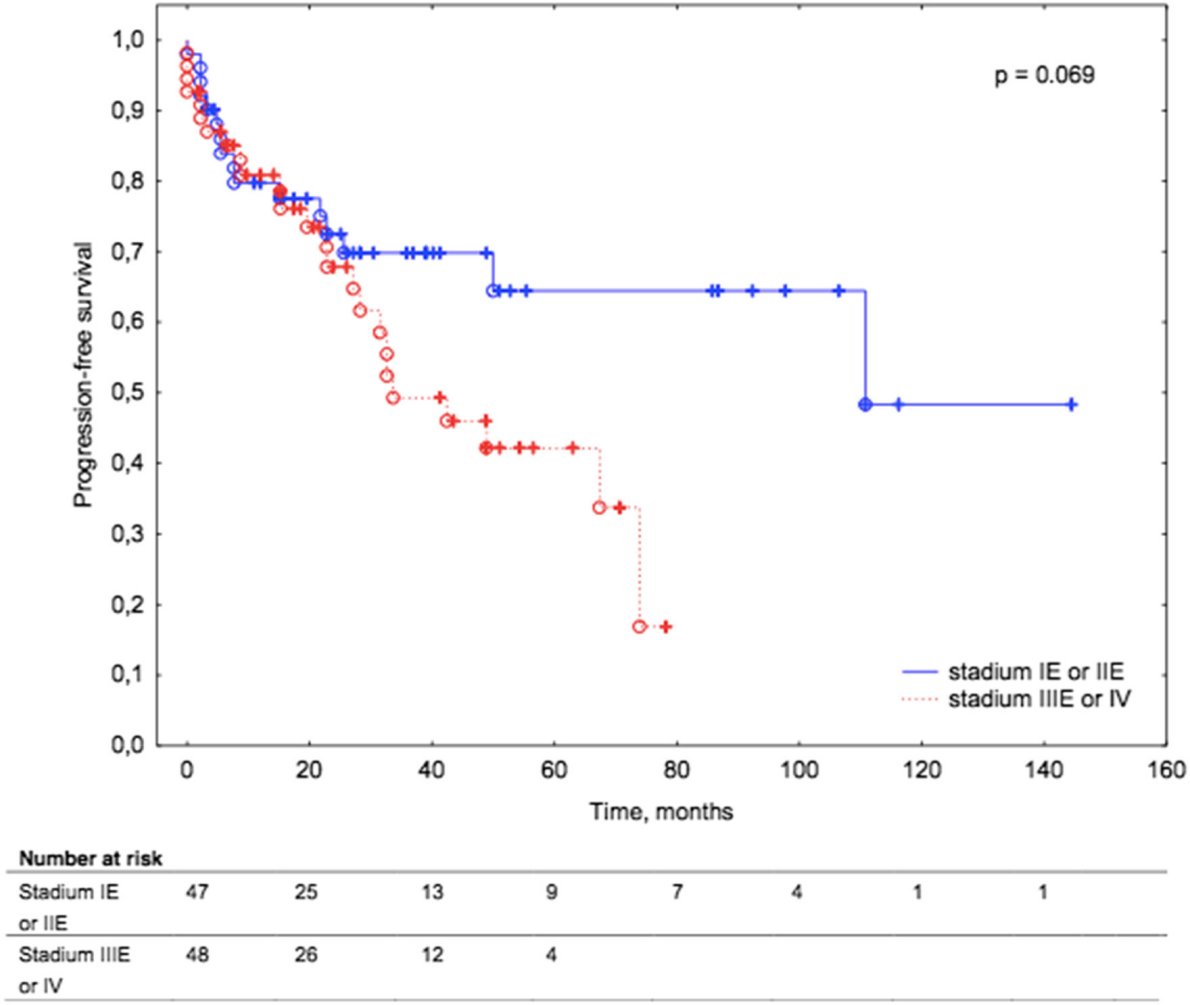
Kaplan–Meier survival curves for progression‐free survival in patients with primary ocular adnexal lymphoma versus secondary ocular adnexal lymphoma.

The median PFS across patients with MALT lymphoma was not reached. The median PFS across non‐MALT lymphoma was 32,7 months. Patients with non‐MALT lymphoma had a significantly inferior PFS compared to patients with MALT lymphoma, (*p* = 0.047; Figure 1). Among 55 patients with MALT lymphoma and 52 patients with non‐MALT lymphoma, progression was reported in 15 (27%) and 26 (50%) cases, respectively (*p* = 0.047). Patients with primary orbital lymphoma had a superior PFS compared to patients with secondary orbital lymphoma [median PFS 104.5 months vs. 33.4 months], (*p* = 0.069; Figure 2).

**FIGURE 3:**
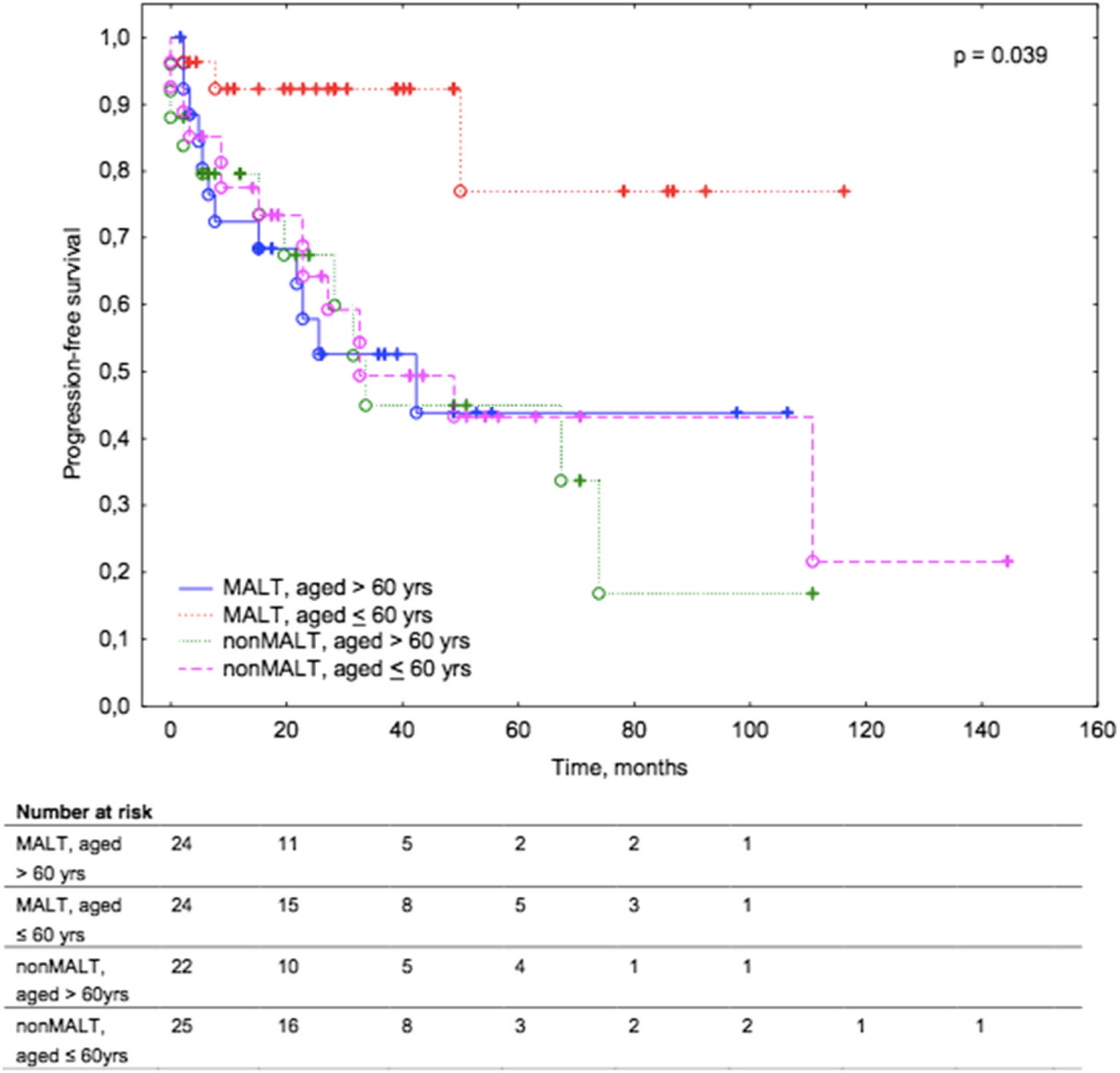
Kaplan–Meier survival curves for progression‐free survival in patients with ocular adnexal lymphoma according to lymphoma subtype (MALT vs non‐MALT) and age (<60 years vs >60 years).

Younger patients with MALT lymphoma were characterized by superior PFS (median PFS not reached) compared to other studied subgroups of patients (older patients with MALT lymphoma, younger and older non‐MALT lymphoma patients) with a median PFS of 30.5, 32.2, 32.6 months, respectively (*p* = 0.039; Figure 3).

Across all studied patients, the median OS was not reached. There were no differences between patients with MALT versus patients with non‐MALT lymphoma regarding overall survival. Among patients with MALT lymphoma, older patients (with the age >60 years) had a significantly inferior OS compared to the younger patients (with the age <60 years) [median OS not reached in any sub‐group].

**FIGURE 4:**
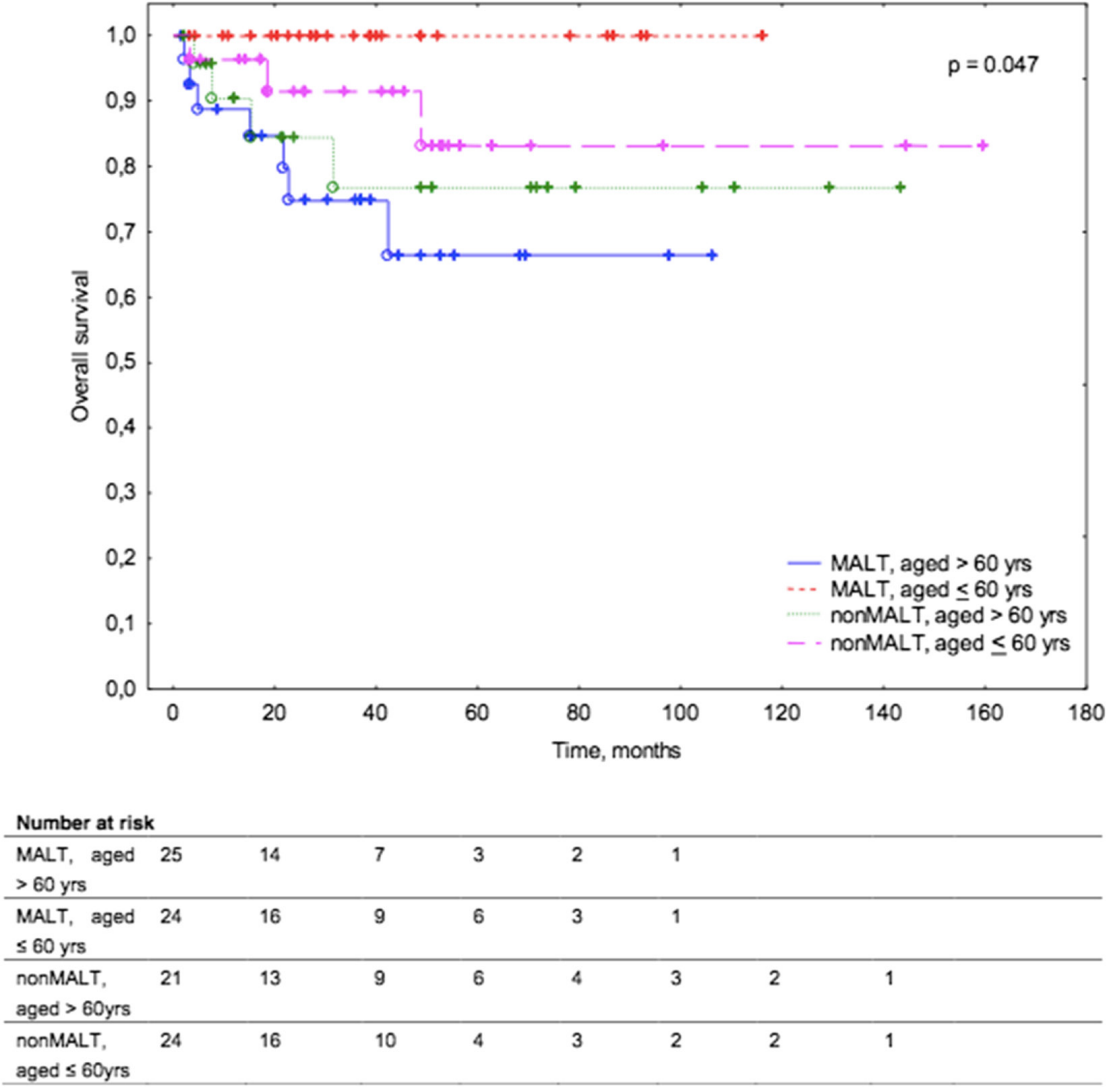
Kaplan–Meier survival curves for overall survival in patients with ocular adnexal lymphoma according to Lymphoma subtype (MALT vs non‐MALT) and age (<60 years vs >60 years).

During over 8 years of observation, among older patients with MALT lymphoma, seven deaths were reported, whereas among younger MALT lymphoma patients no death was reported (*p* = 0.047; Figure 4).

**FIGURE 5:**
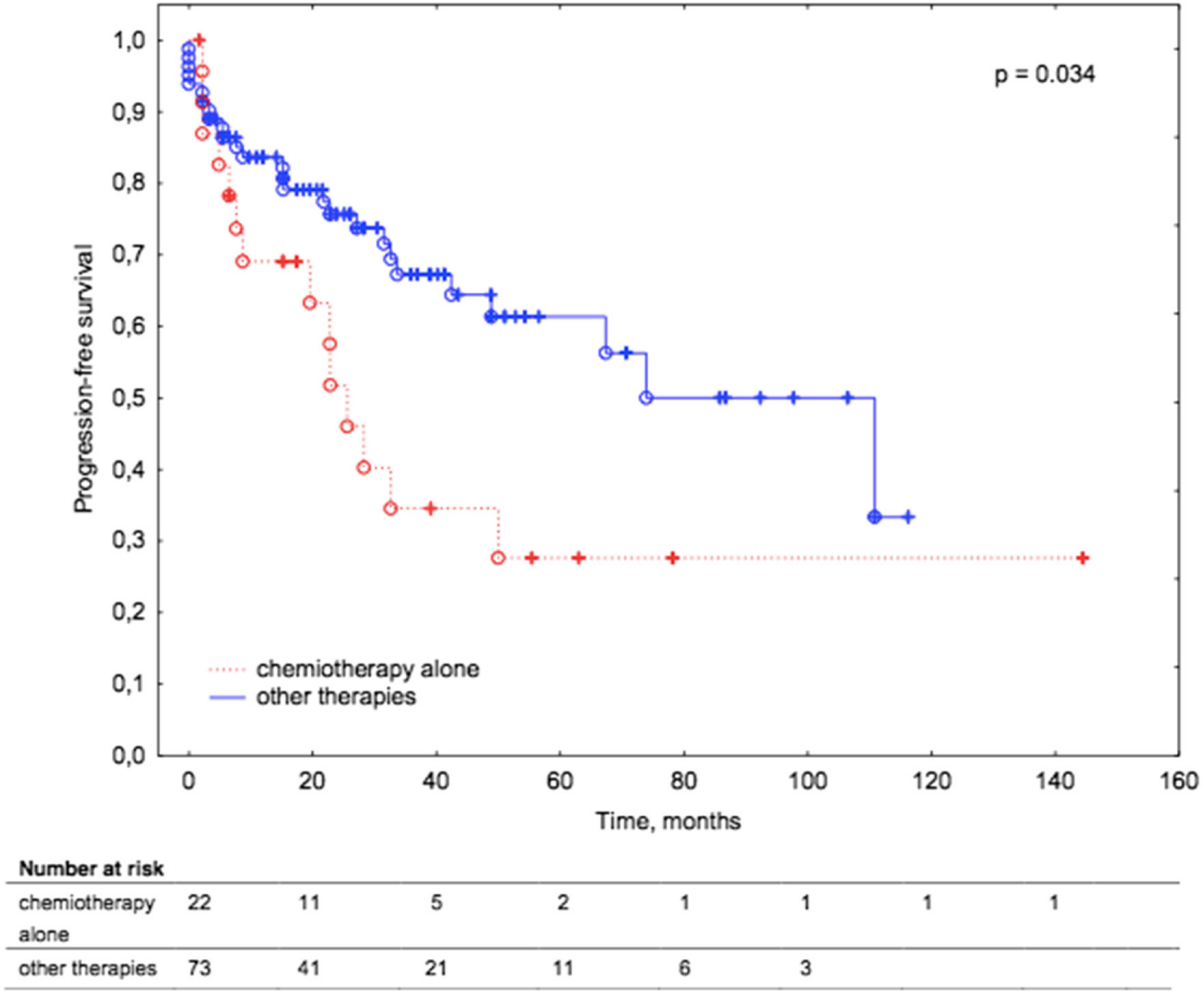
Kaplan–Meier survival curves for progression‐free survival in patients with ocular adnexal lymphoma according to treatment modality (chemotherapy alone vs combined therapy).

Regarding first‐line treatment, 33 patients (31%) were treated with immunochemotherapy. Twenty‐five patients (23%) received systemic chemotherapy alone. Surgery alone was performed in 12 patients (11%) and in combination with chemotherapy in six patients (6%). Radiotherapy alone was applied in seven patients (7%) and in combination with chemotherapy in 18 patients (17%). Another two patients were treated with combination of surgery and radiotherapy or immunotherapy alone. No association was found between overall survival and applied treatment modalities. Regarding PFS, patients treated with chemotherapy alone had inferior PFS compared to patients treated with combined therapies or surgery (*p* = 0.034; Figure 5). The median PFS across patients received chemotherapy alone was 23.7 months, whereas across other patients was 73.9 months. 56% of patients treated with chemotherapy alone experienced disease progression, whereas only 33% of patients received combined treatments or surgery or radiotherapy had lymphoma progression.

In multivariate models for PFS, older age, the presence of proptosis and treatment only based on chemotherapy, as well as non‐MALT histopathological types were identified to adversely affect PFS (Table 3).

**TABLE 3:** Univariate and multivariate analysis for PFS in patients with orbital lymphoma

Variable	Univariate		Multivariate	
	HR (95% CI)	*p*	HR (95% CI)	*p*
Age [years]	1.02 (1.00–1.05)	0.035	1.03 (1.00–1.05)	0.019
Chemotherapy alone	2.02 (1.06–3.85)	0.034	1.93 (0.98–3.79)	0.057
MALT subtype	0.53 (0.28–1.01)	0.053	0.51 (0.27–0.97)	0.039
Proptosis	1.89 (0.89–4.01)	0.097	2.26 (1.02–5.01)	0.044

## 4 DISCUSSION

In our multicenter study, in which eight Polish hematological centers affiliated with PLRG participated, 107 patients with orbital lymphoma were included. This retrospective analysis investigated the clinical characteristics of orbital lymphoma, as well as the survival outcome. Currently, our study is the largest reported data of patients with orbital lymphoma in Central and Eastern Europe.

We found only a slightly MALT lymphoma predominance in general orbital lymphoma which is consistent with previous reports.^2^, ^11^ However, in other studies, especially from Asia, the incidence of MALT lymphoma was significantly higher, ranging from 66% to 82% of total orbital lymphoma.^1^, ^3^, ^12^, ^13^ Unexpectedly, among non‐MALT lymphoma, almost 30% of patients had diffuse large‐cell lymphoma. Only 7% of patients had mantle cell lymphoma and 4% of patients had follicular lymphoma. Both DLBCL, MCL, and FL were diagnosed in advanced stage with systemic involvement. The influence of geographic differences is significant in the context of the prevalence of particular histological subtypes of ocular adnexal lymphoma.^1^, ^4^, ^8^, ^14^ A relatively high total percentage of non‐MALT lymphoma in our report, including aggressive lymphomas, may indicate a new pattern of distribution of orbital adnexal lymphoma histological subtypes in the population of Central and Eastern Europe.

In our study, most patients with B‐cell orbital lymphoma were female (64%). A female predominance was present among MALT and FL which is consistent with previous data.^15^ Our study revealed also higher incidence of MCL in females which is opposite to other reports.^8^, ^9^, ^10^, ^16^, ^17^ T/NK cell orbital lymphoma exhibited male predominance (83%) similarly to other studies.^15^

The median age in our study was 60 years which is lower compared to reports from Western countries.^2^ Among B‐cell orbital lymphoma, the median age was lower in patients with MCL and FL subtype (56 years and 54 years, respectively). Previous studies reported higher median age among patients with MCL compared to other subtypes.^2^, ^15^ The youngest patients with a median age of 47 years had T/NK cell orbital lymphoma which is consistent with other studies.^15^

At the time of diagnosis, the majority of patients with MALT lymphoma were Ann Arbor stage IE/IIE which is consistent with previous studies.^2^ Among non‐MALT lymphoma patients, only 16% accounted for primary lymphoma. The majority of patients with DLBCL (80%) and all patients with FL and MCL had secondary manifestation. The most important result from our study was the fact that almost half of the patients with orbital lymphoma had secondary manifestation (stage IIIA and IV) and two‐thirds of the patients did not have localized disease and were characterized by clinical stage higher than IE such as IIE, IIIE and/or IV. The high incidence of advanced stages of orbital lymphoma in our study can be explained by the collection of data in large hematological centers, where each patient was screened for systemic disease upon admission.

Similar results were reported in previous study which emphasized the role of broad staging procedures in patients admitted with orbital lymphoma.^4^

In our study more than half of the patients were treated with combined therapies, including 31% of the patients received immunochemotherapy, 17% of the patients received radiotherapy in combination with chemotherapy or immunochemotherapy, and 13% of the patients underwent surgery in combination with immunotherapy (monoclonal antibody against CD20 – rituximab) or chemotherapy. We demonstrated that patients who received combined treatment had superior PFS compared to patients treated with chemotherapy alone. In our study, we confirmed a correlation between poorer PFS and chemotherapy treatment (HR = 1.93, *p* = 0.057). In our cohort, due to high percentage of patients with systemic involvement of orbital adnexal lymphoma and high‐grade lymphomas, treatment with chemotherapy alone as the primary treatment modality concerned almost one‐fourth of the patients. Besides, chemotherapy treatment as the primary modality was chosen in our study in some cases of low‐grade MALT lymphoma, when the tumor was in close localization to vital ocular adnexa as in other studies.^18^ This approach is consistent with previous reports where chemotherapy was used in MALT lymphoma because of the closeness of the tumor to the eye and allowed to avoid violation of the integrity and function of the ocular adnexa.^18^ Importantly, in our study, considering all the histological subtypes of lymphoma, including advanced and aggressive ones, chemotherapy which is a standard treatment modality in case of aggressive lymphoma, was unsatisfactory in the terms of PFS. The addition of radiotherapy, monoclonal antibody, or surgery improved PFS in patients with orbital adnexal lymphoma. Therefore, our study highlights the key role of combined therapies including monoclonal antibodies in overall population of orbital lymphoma patients.

One of the major findings in our study was poorer PFS in the whole group of non‐MALT lymphoma patients compared to MALT lymphoma patients. Patients with MALT lymphoma had a twice lower risk of progression compared to patients suffered from other histological subtypes of lymphoma (HR = 0.51, *p* = 0.039). Moreover, patients with primary orbital lymphoma had a superior PFS compared to patients with secondary orbital lymphoma.

We demonstrated that the recurrence rate was related to age (HR = 1.03, *p* = 0.0019). The original MALT‐lymphoma International Prognostic Index (MALT‐IPI) considers age >70 years as a strong prognostic factor.^19^ In our study, the MALT lymphoma patient population was younger, with a median age of 62 years. Even so, age was an indicator of progression‐free survival. Results from our study showed that patients with MALT lymphoma who had <60 years were characterized by superior PFS compared to other studied subgroups of patients (older patients with MALT lymphoma, both younger and older non‐MALT lymphoma patients).

Our study showed one more interesting result ‐ patients with proptosis had a more than two times higher risk of progression (HR = 2.26, *p* = 0.044). This symptom might reflect the tumor volume and muscle infiltration, which has been associated with poor prognosis in previous studies.^20^, ^21^

There were some limitations in our report. First, due to its retrospective nature, there was a lack of complete data on the location and laterality of the lymphoma as well as the results of laboratory findings and therefore these data could not be included in the analysis. Second, we had no information on possible pathogenesis factors such as Helicobacter pylori, Chlamydia psittaci, HCV which limited the analysis of the potential relationship between these factors and the occurrence of specific subtypes of ocular lymphoma in our study population.

## 5 CONCLUSIONS

We have demonstrated a significant percentage of secondary orbital lymphomas in the population of Polish patients. MALT lymphomas accounted for just over half of the overall population of patients with orbital lymphomas. Among MALT lymphoma, one‐third of the patients did not have localized disease and were characterized by clinical stage higher than stage IE.

Due to the high prevalence of advanced stages of orbital lymphoma, combined treatment of orbital lymphoma is warranted. Treatment with combined therapies was associated with superior PFS compared to chemotherapy alone in the overall population of patients with orbital lymphoma. Finally, we determined prognostic factors which adversely affect the PFS such as age, non‐MALT histopathological diagnosis, and the presence of proptosis.

## AUTHOR CONTRIBUTIONS

**Elżbieta Kalicińska:** Conceptualization (lead); data curation (equal); formal analysis (equal); investigation (equal); methodology (equal); visualization (equal); writing – original draft (lead). **Agnieszka Giza:** Investigation (equal); resources (equal). **Jan Maciej Zaucha:** Resources (equal); supervision (equal). **Sebastian Giebel:** Resources (equal); supervision (equal). **Dagmara Zimowska‐Curyło:** Resources (equal). **Iga Andrasiak:** Data curation (equal); formal analysis (equal); validation (lead). **Wojciech Spychałowicz:** Resources (equal). **Jerzy Wojnar:** Resources (equal); supervision (equal). **Andrzej Balcerzak:** Resources (equal). **Joanna Romejko‐Jarosinska:** Resources (equal). **Ewa Paszkiewicz‐Kozik:** Resources (equal). **Wanda Knopińska‐Posłuszny:** Resources (equal). **Justyna Rybka:** Resources (equal); supervision (equal). **Paula Jabłonowska:** Visualization (equal). **Tomasz Wrobel:** Conceptualization (supporting); methodology (equal); supervision (equal); writing – review and editing (lead).

## Funding information

This research received no external funding.

## CONFLICT OF INTEREST

The authors declare no conflict of interest.

## INSTITUTIONAL REVIEW BOARD STATEMENT

The study was conducted according to the guidelines of the Declaration of Helsinki, and approved by the Institutional Ethics Committee of Wroclaw Medical University (protocol code no 315/2020).

## INFORMED CONSENT STATEMENT

Collected data did not include any personally identifiable information therefore informant consents from the patients were not required.

## Data Availability

The data are available from the corresponding author.
